# Supplementary material for: NLRC5 inhibits neointima formation following vascular injury and directly interacts with PPARγ
Source: Nat Commun. 2019 Jun 28;10:2882. doi: 10.1038/s41467-019-10784-y (PMC6599027; doi:10.1038/s41467-019-10784-y)
Supplement: Supplementary file 1 — Supplementary Information [file 41467_2019_10784_MOESM1_ESM.pdf]

Supplementary Information

NLRC5 inhibits neointima formation following vascular injury and directly interacts with PPARγ

Luan et al.

Table of Contents

Supplementary Figure 1. NLRC5 is abundant in coronary artery from the patient with Kawasaki.

Supplementary Figure 2. NLRC5 is more abundant in coronary plaque compared with normal coronary artery.

Supplementary Figure 3. NLRC5 is analogously expressed in the endothelium of sham and ligated carotid arteries in C57BL/6 mice.

Supplementary Figure 4. NLRC5 is expressed in carotid arteries of $Nlrc5^{+/+}$ mice, but not in $Nlrc5^{-/-}$ mice.

Supplementary Figure 5. Deficiency of *Nlrc5* does not affect systolic blood pressure (SBP), heart rate and metabolic parameters in mice fed with normal chow.

Supplementary Figure 6. Depletion of *Nlrc5* reduces the proportion of $CD8^+$ T cells in spleen and peripheral blood.

Supplementary Figure 7. Depletion of *Nlrc5* does not influence the proportion of myeloid cells in bone marrow, spleen and peripheral blood.

Supplementary Figure 8. Deficiency of *Nlrc5* does not affect the recruitment of CD45 positive cells into the ligated carotid arteries.

Supplementary Figure 9. Overexpression of NLRC5 by local transduction of *Nlrc5* adenoviruses is confirmed by immunofluorescence staining.

Supplementary Figure 10. Overexpression of NLRC5 alleviates VSMC proliferation, migration and dedifferentiation.

Supplementary Figure 11. Deficiency of *NLRC5* does not affect the apoptosis of smooth muscle cells after carotid ligation or starvation.

Supplementary Figure 12. Decreased *NLRC5* does not affect inflammation and HLA expression in VSMCs.

Supplementary Figure 13. Smad2 phosphorylation is stimulated by high glucose but not by PDGF-BB in HASMCs.

Supplementary Figure 14. Effects of pioglitazone and T0070907 on PPARγ expression and activity.

Supplementary Figure 15. Effects of pioglitazone and T0070907 on HASMC phenotype.

Supplementary Figure 16. Identification of C57BL/6 recipient harboring GFP transgenic bone marrow cells by flow cytometry.

Supplementary Figure 17. Lethally-irradiated wild-type (WT) mice received bone marrow transplantation (BMT) from WT or *Nlrc5*$^{-/-}$ (KO) donors followed by 3 weeks recovery.

Supplementary Table 1. The baseline characteristics of the patients.
Supplementary Table 2. Summary of fluorescently labelled
Supplementary Table 3. Summary of primer sequences used for RT-PCR and ChIP.

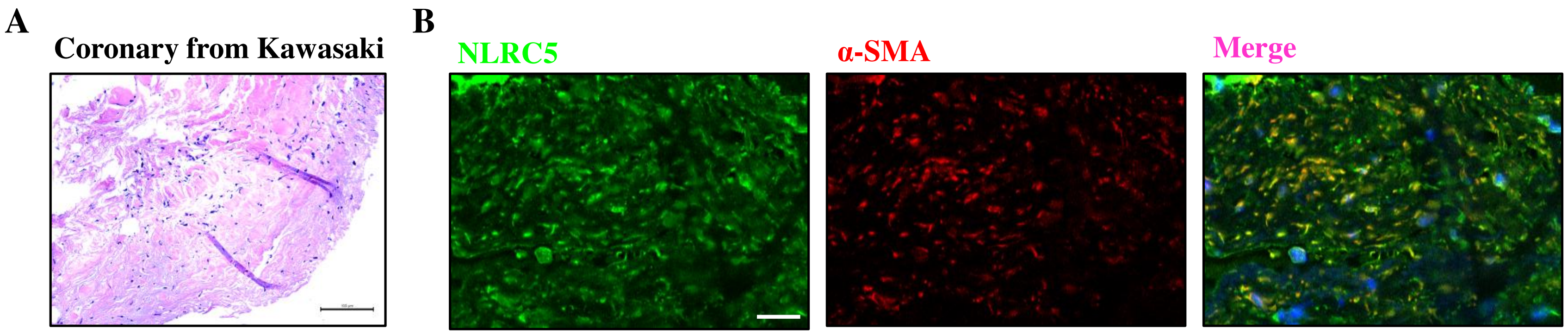

Supplementary Figure 1. NLRC5 is abundant in coronary artery from the patient with Kawasaki.
A. Representative images of hematoxylin/eosin-stained human coronary artery from Kawasaki patient. Scale bar: 100 µm. **B.** Immunofluorescence staining shows that NLRC5 (green) is constitutively expressed in the coronary from Kawasaki patient. Scale bar: 20 µm.

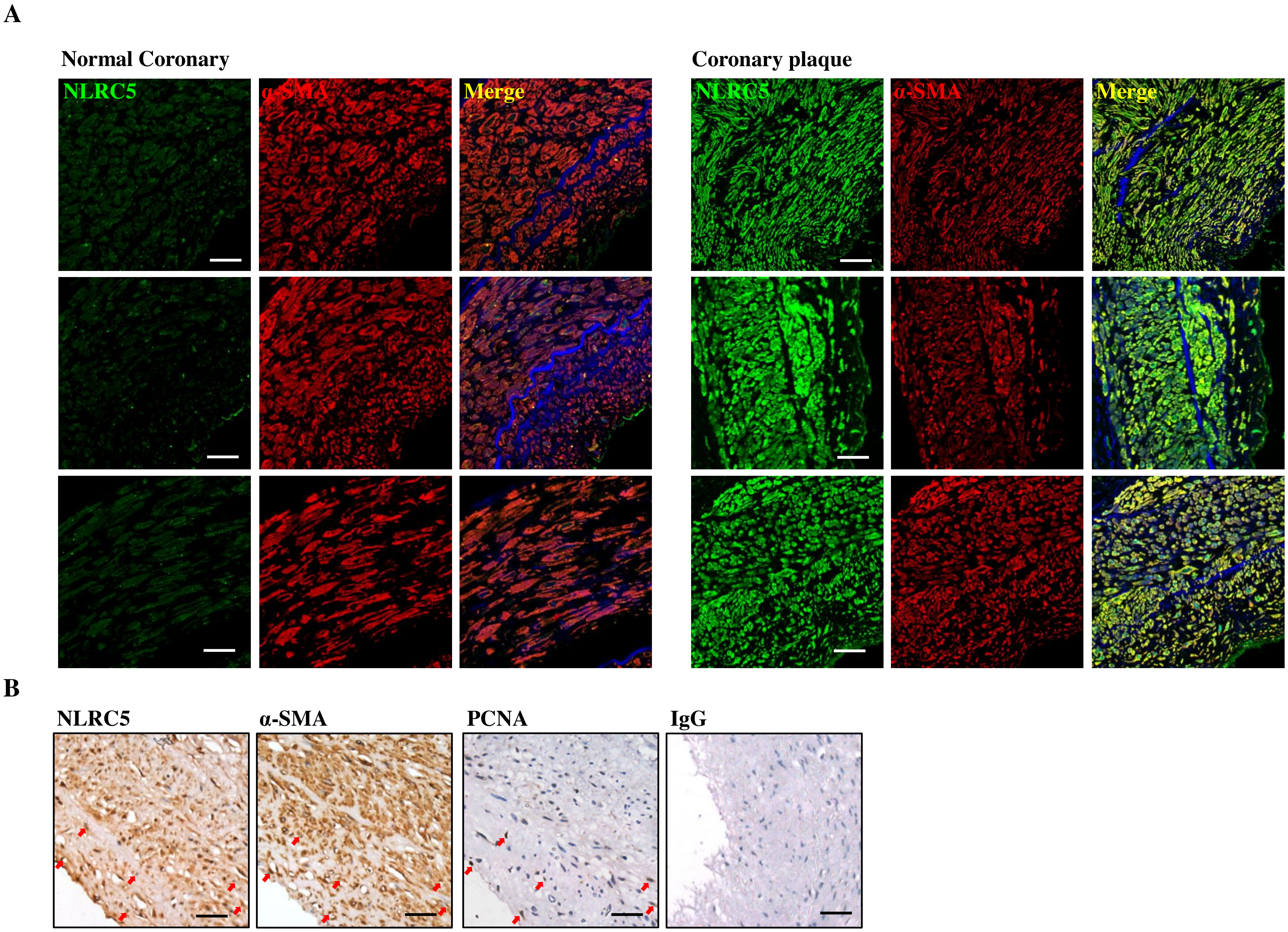

Supplementary Figure 2. NLRC5 is more abundant in coronary plaque compared with normal coronary artery.
A. Immunofluorescence staining of NLRC5 (green), α-SMA (red) and DAPI (blue) in human coronary plaque and normal coronary artery (n=3 per group). Scale bar: 20 µm. **B.** Immunohistochemistry staining with NLRC5, α-SMA and PCNA in series sections of human coronary plaques. Scale bar: 20 µm.

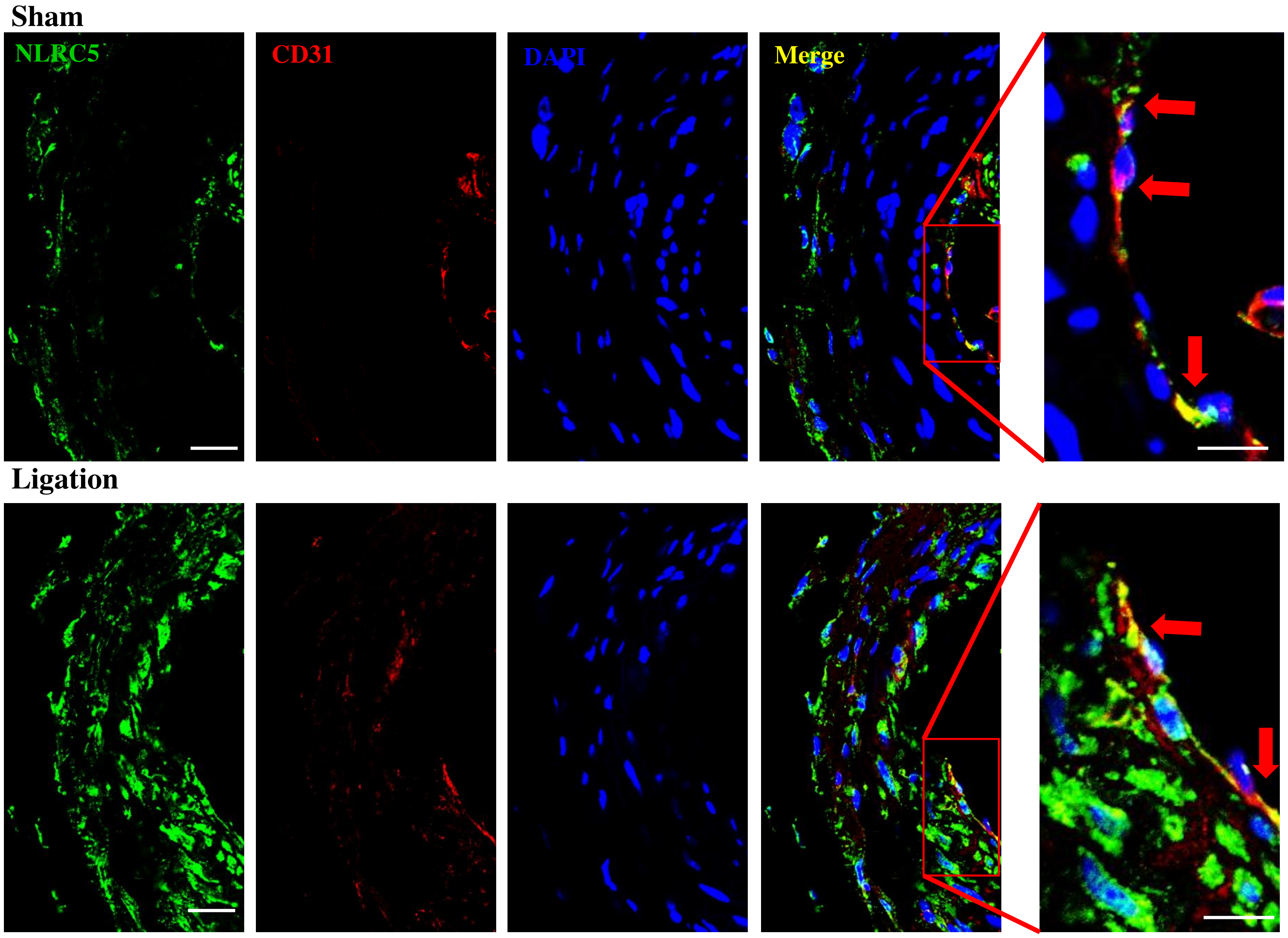

Supplementary Figure 3. NLRC5 is analogously expressed in the endothelium of sham and ligated carotid arteries in C57BL/6 mice. Endothelial cells are immunostained with anti-CD31 antibodies (red) and Nlrc5 is shown in green (n=3 per group). Scale bar: 50 μm (left) and 20 μm (right). Source data are provided as a Source Data file.

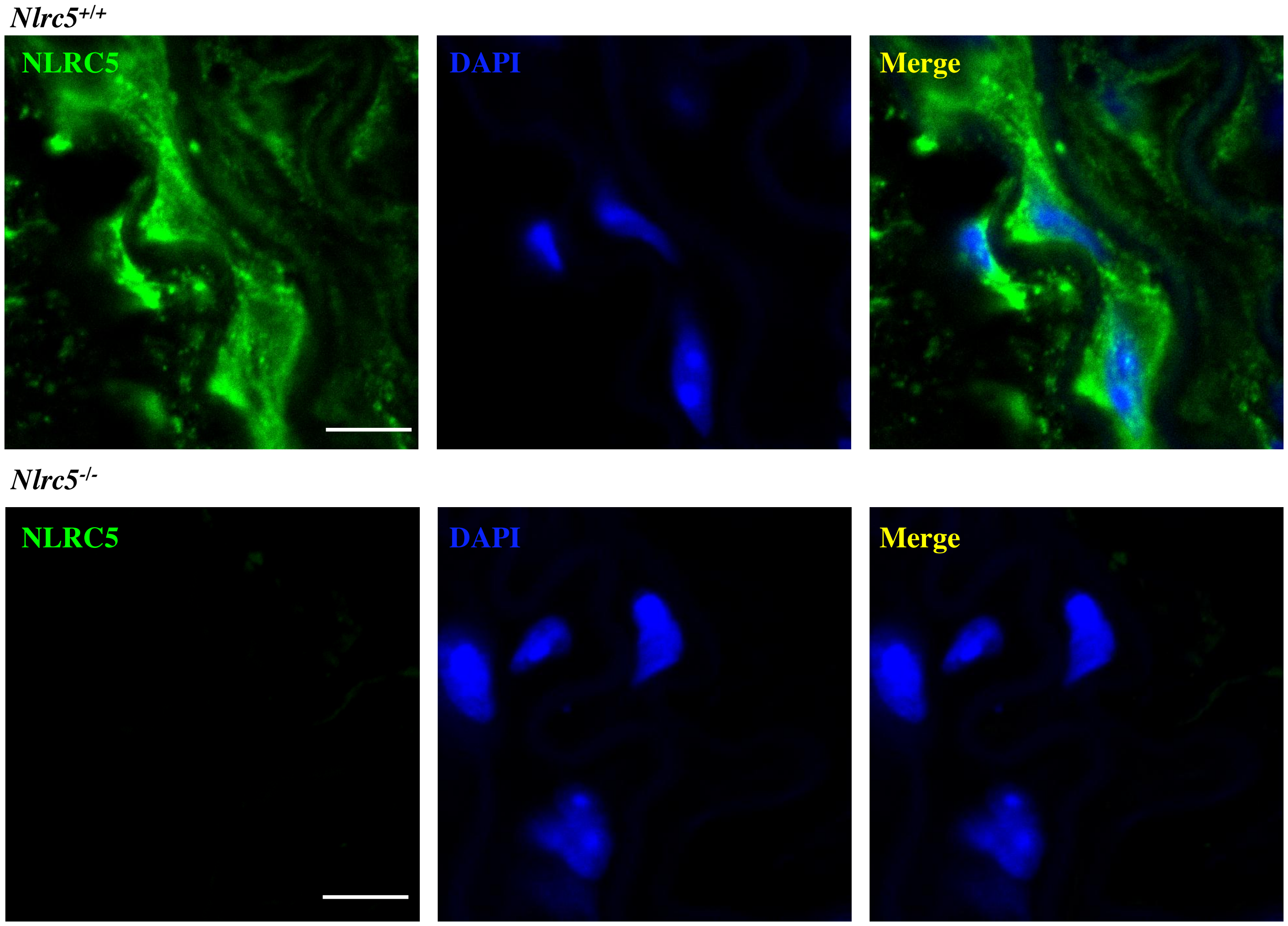

Supplementary Figure 4. NLRC5 is expressed in carotid arteries of *Nlrc5*$^{+/+}$ mice, but not in *Nlrc5*$^{-/-}$ mice.

Immunofluorescence staining of Nlrc5 (green) and DAPI (blue) in carotid arteries from *Nlrc5*$^{+/+}$ and *Nlrc5*$^{-/-}$ mice (n=3 per group). Scale bar: 20 µm.

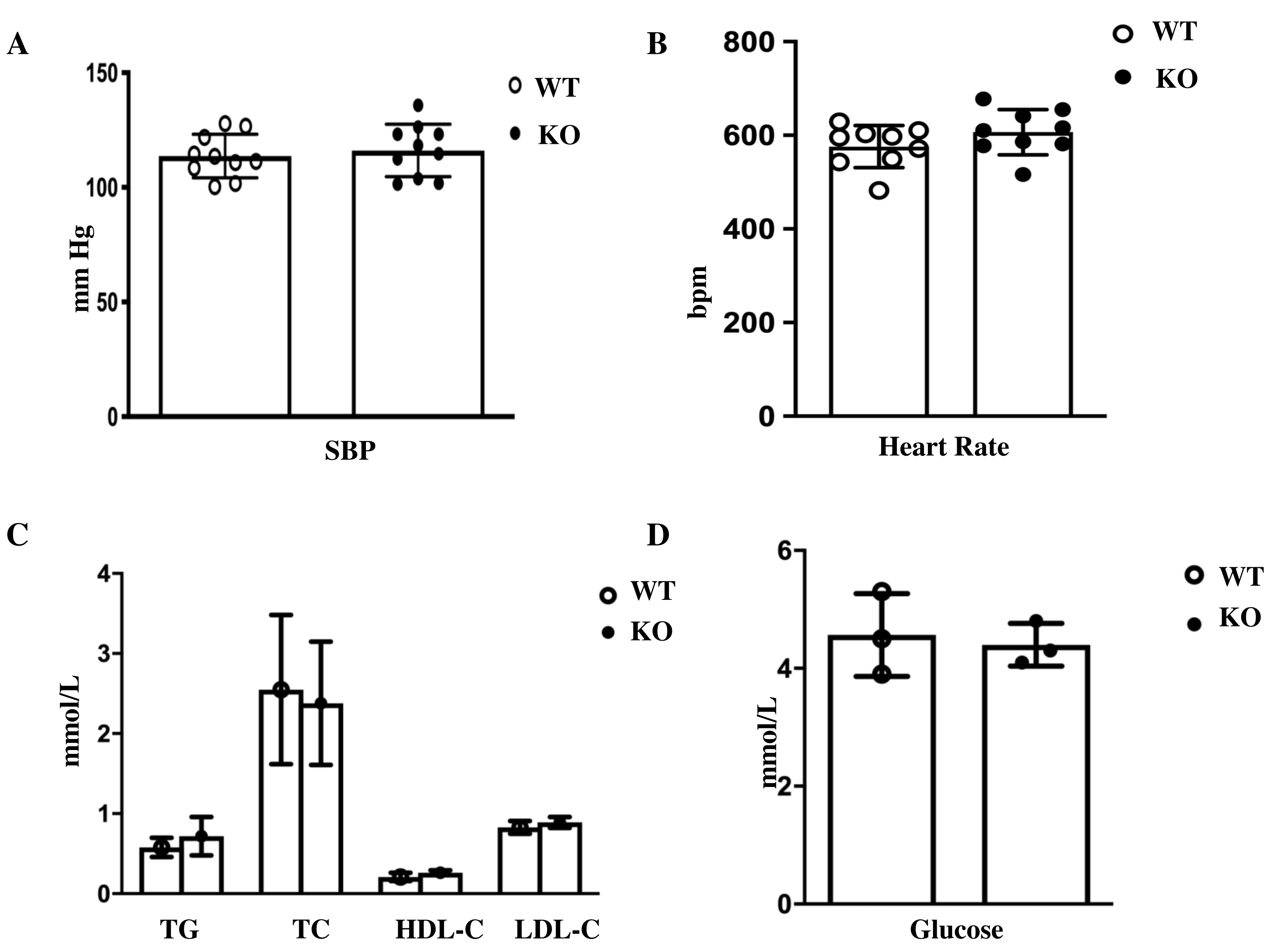

Supplementary Figure 5. Deficiency of *Nlrc5* does not affect systolic blood pressure (SBP), heart rate and metabolic parameters in mice fed with normal chow.
A. Noninvasive tail cuff monitoring of systolic BP of *Nlrc5*$^{+/+}$ (WT) and *Nlrc5*$^{-/-}$ (KO) mice at 3 weeks after carotid ligation. **B.** Noninvasive tail cuff monitoring of heart rate of WT and KO mice at 3 weeks after carotid ligation. **C.** Plasma levels of total triglyceride (TG), total cholesterol (TC), low density lipoprotein cholesterol (LDL-C) and high density lipoprotein (HDL-C) of WT and KO mice at 3 weeks after carotid ligation. **D.** Plasma levels of fasting glucose of WT and KO mice at 3 weeks after carotid ligation. (n=7 per). Two-tailed Student's t-test was used to compare two groups. Data are presented as mean ± SD. * *P*<0.05. Source data are provided as a Source Data file.

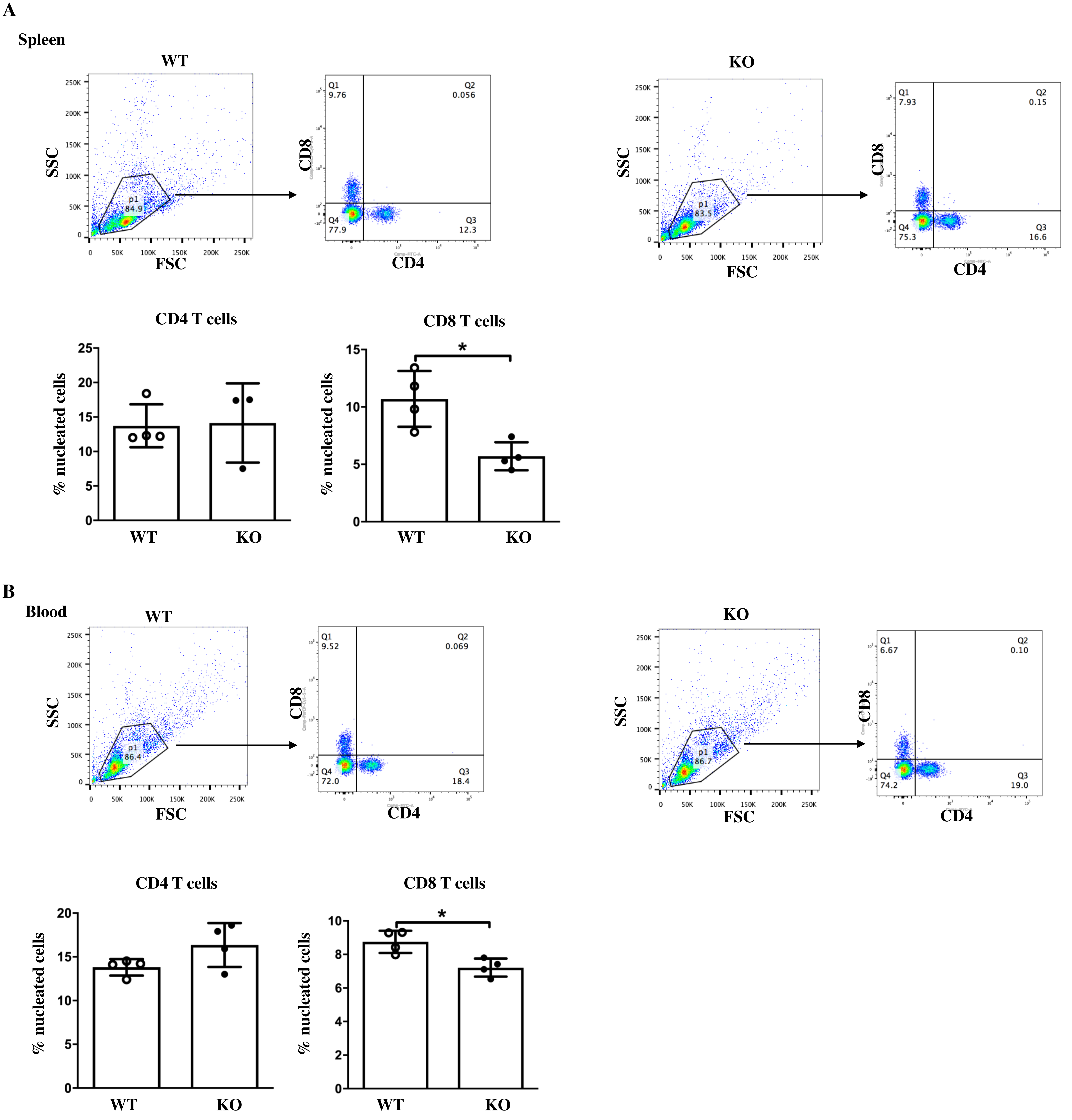

Supplementary Figure 6. Depletion of *Nlrc5* reduces the proportion of CD8$^+$ T cells in spleen and peripheral blood.
Gating strategy to determine the percentage of CD8$^+$ T cells and CD4$^+$ T cells in spleen (**A**) and peripheral blood (**B**). Depletion of *Nlrc5* significantly reduces the proportion of CD8$^+$ T cells but not alters the proportion of CD4$^+$ T cells in spleen and peripheral blood at 3 weeks after carotid ligation. (n=7 per group). Two-tailed Student's t-test was used to compare two groups. Data are presented as mean ± SD. WT = *Nlrc5*$^{+/+}$, KO = *Nlrc5*$^{-/-}$. *P<0.05. Source data are provided as a Source Data file.

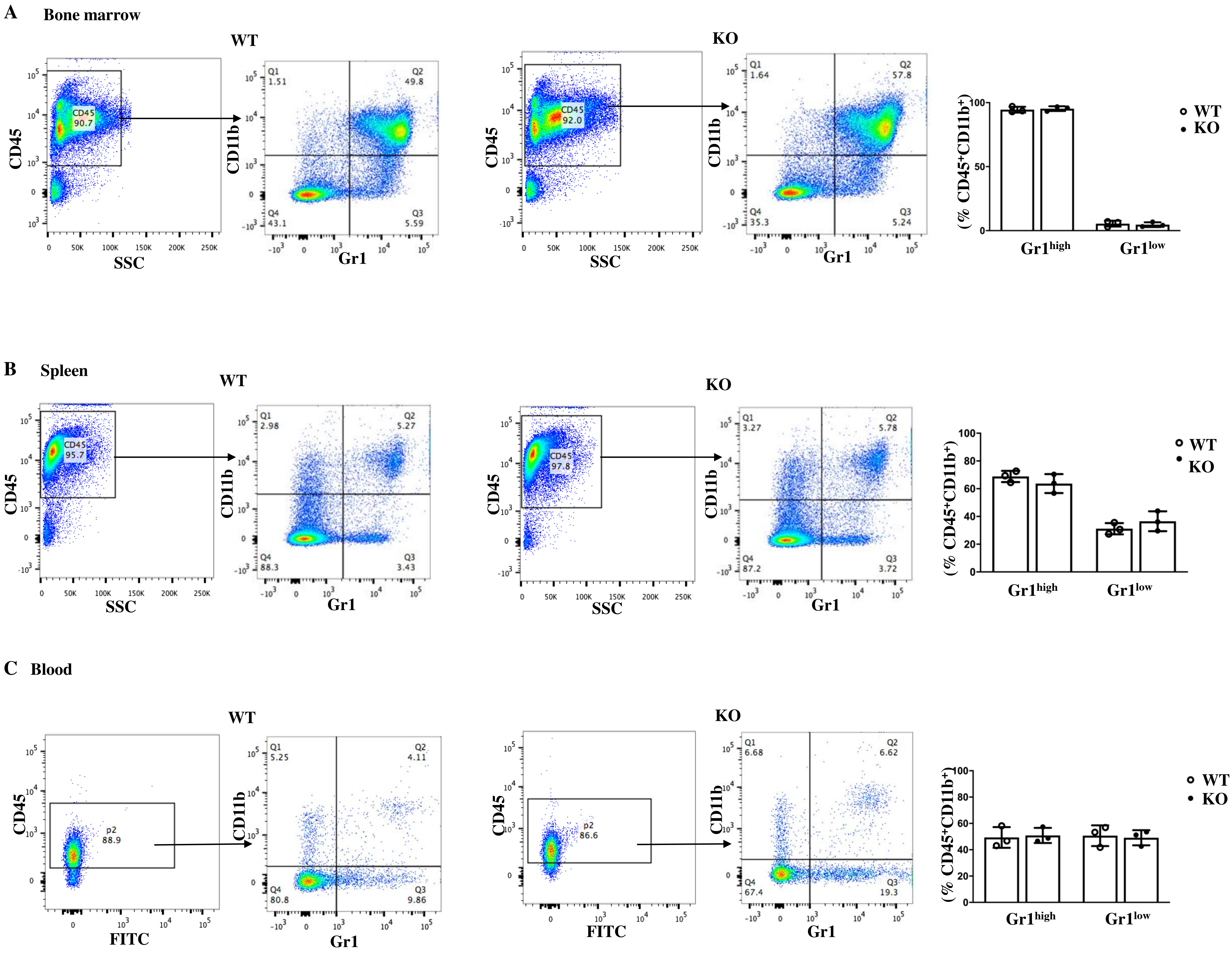

Supplementary Figure 7. Depletion of *Nlrc5* does not influence the proportion of myeloid cells in bone marrow, spleen and peripheral blood.
Gating strategy to determine the percentage of CD11b$^+$ cells and Gr1$^+$ cells in bone marrow (**A**), spleen (**B**) and peripheral blood (**C**). No differences in the proportion of CD45$^+$CD11b$^+$Gr1$^+$ myeloid cells are found in bone marrow **(A)**, spleen **(B)** and peripheral blood **(C)** from WT and KO mice at 3 weeks after carotid ligation. (n=7 per group). Student's t-test was used to compare two groups. Data are presented as mean ± SD. WT= *Nlrc5*$^{+/+}$, KO= *Nlrc5*$^{-/-}$. **P*<0.05. Source data are provided as a Source Data file.

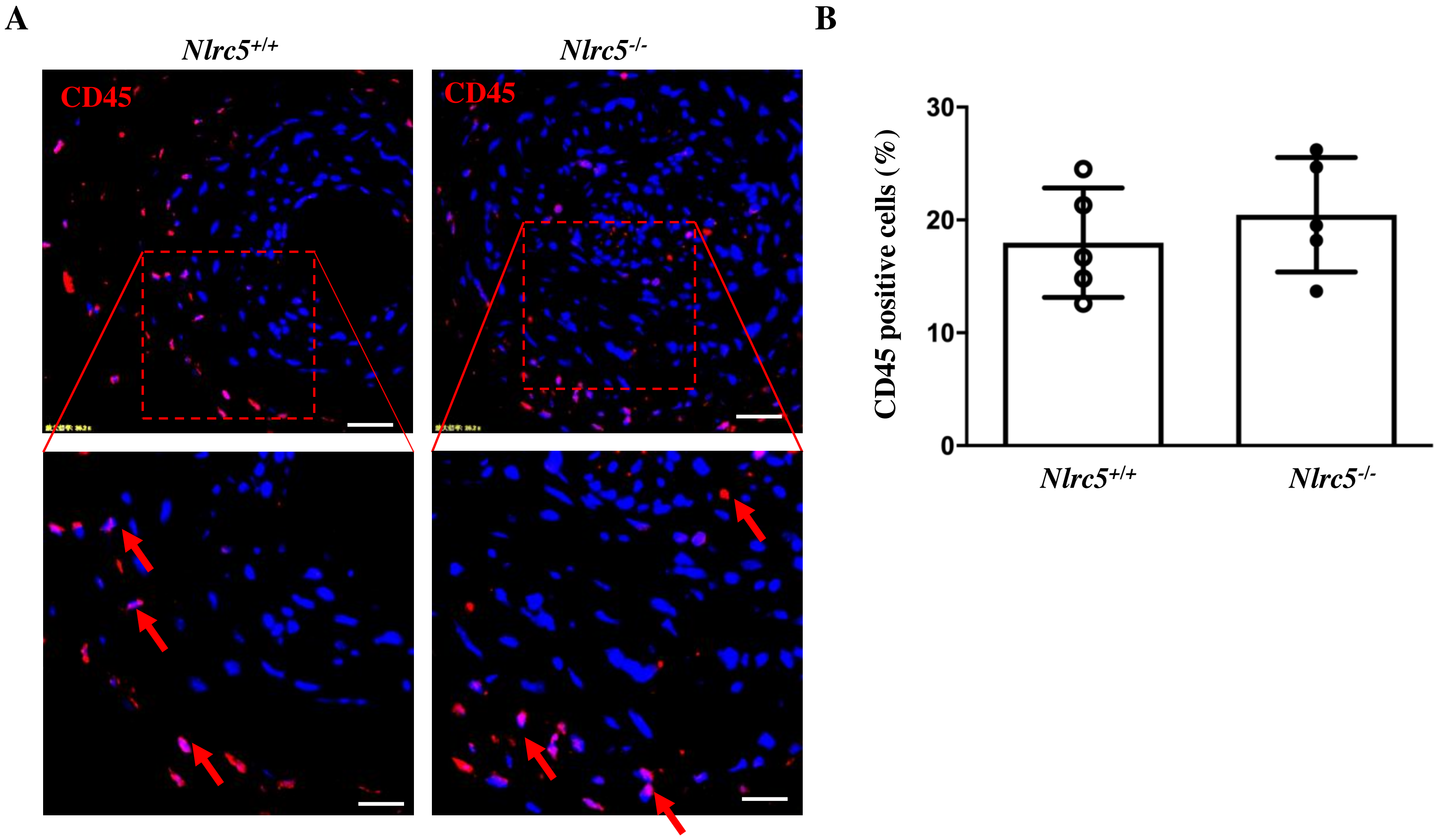

Supplementary Figure 8. Deficiency of *Nlrc5* does not affect the recruitment of CD45 positive cells into the ligated carotid arteries.

A. Representative immunofluorescence images of CD45 positive cells (red arrow) in the ligated carotid arteries of *Nlrc5*$^{-/-}$ and *Nlrc5*$^{+/+}$ mice at 3 weeks after carotid ligation. Scale bar: 50 μm (upper) and 20 μm (lower). **B.** Quantification of the proportion of CD45 positive cells in ligated carotids of *Nlrc5*$^{-/-}$ and *Nlrc5*$^{+/+}$ mice(n=5 per group). Student's t-test was used to compare two groups. Data are presented as mean ± SD. Source data are provided as a Source Data file.

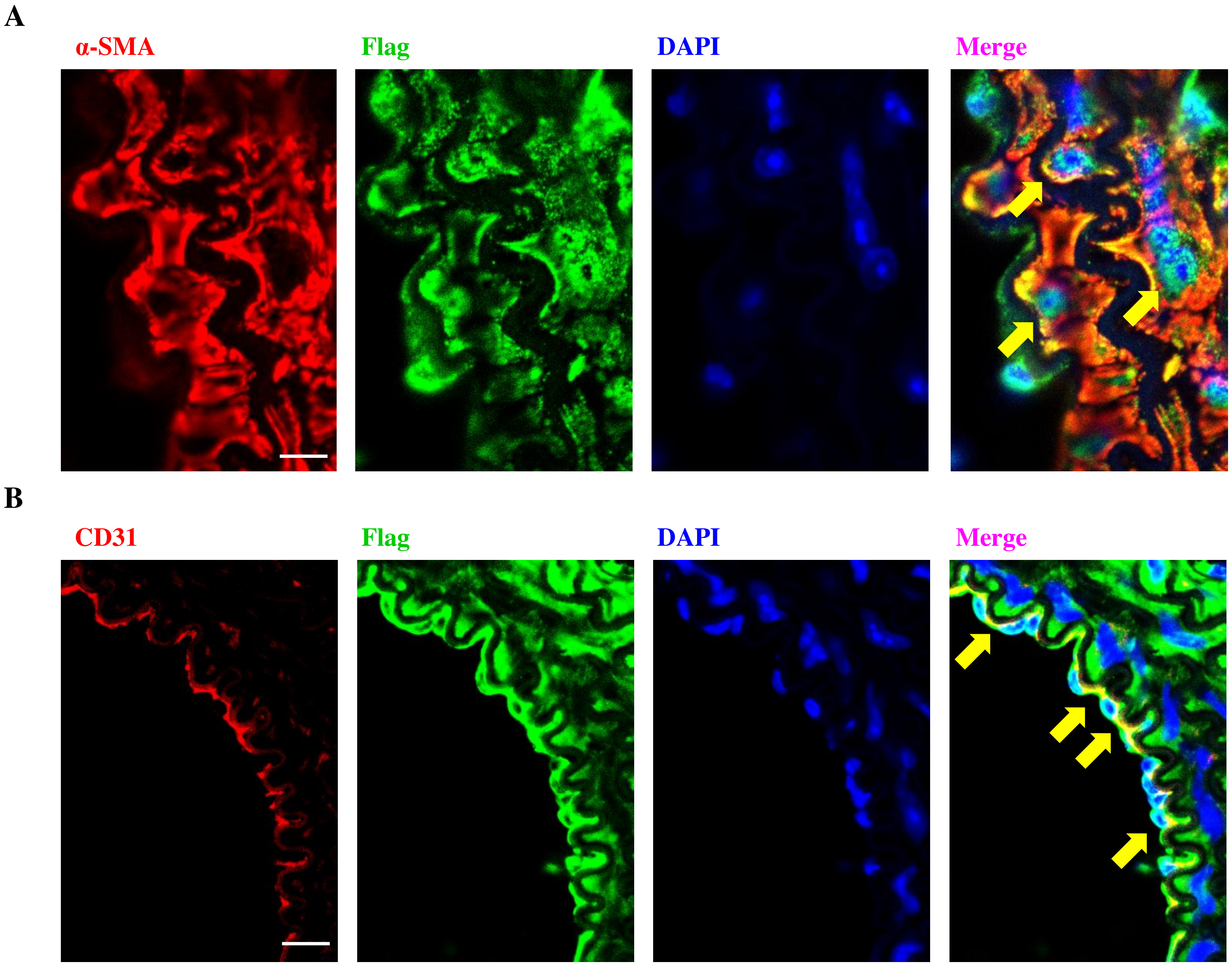

Supplementary Figure 9. Overexpression of NLRC5 by local transduction of *Nlrc5* adenoviruses is confirmed by immunofluorescence staining.

A. Representative immunofluorescence staining depicts that Flag tags (green) are co-located with smooth muscle cells labeled with α-SMA (red) in mouse carotid artery transducted with Flag-tagged Ad-*Nlrc5*. Scale bar: 20 μm. **B.** Representative immunofluorescence staining depicts that Flag tags (green) are co-located with endothelial cells labeled with CD31 (red) in mouse carotid artery transducted with Flag-tagged Ad-*Nlrc5*. Scale bar: 20 μm. Source data are provided as a Source Data file.

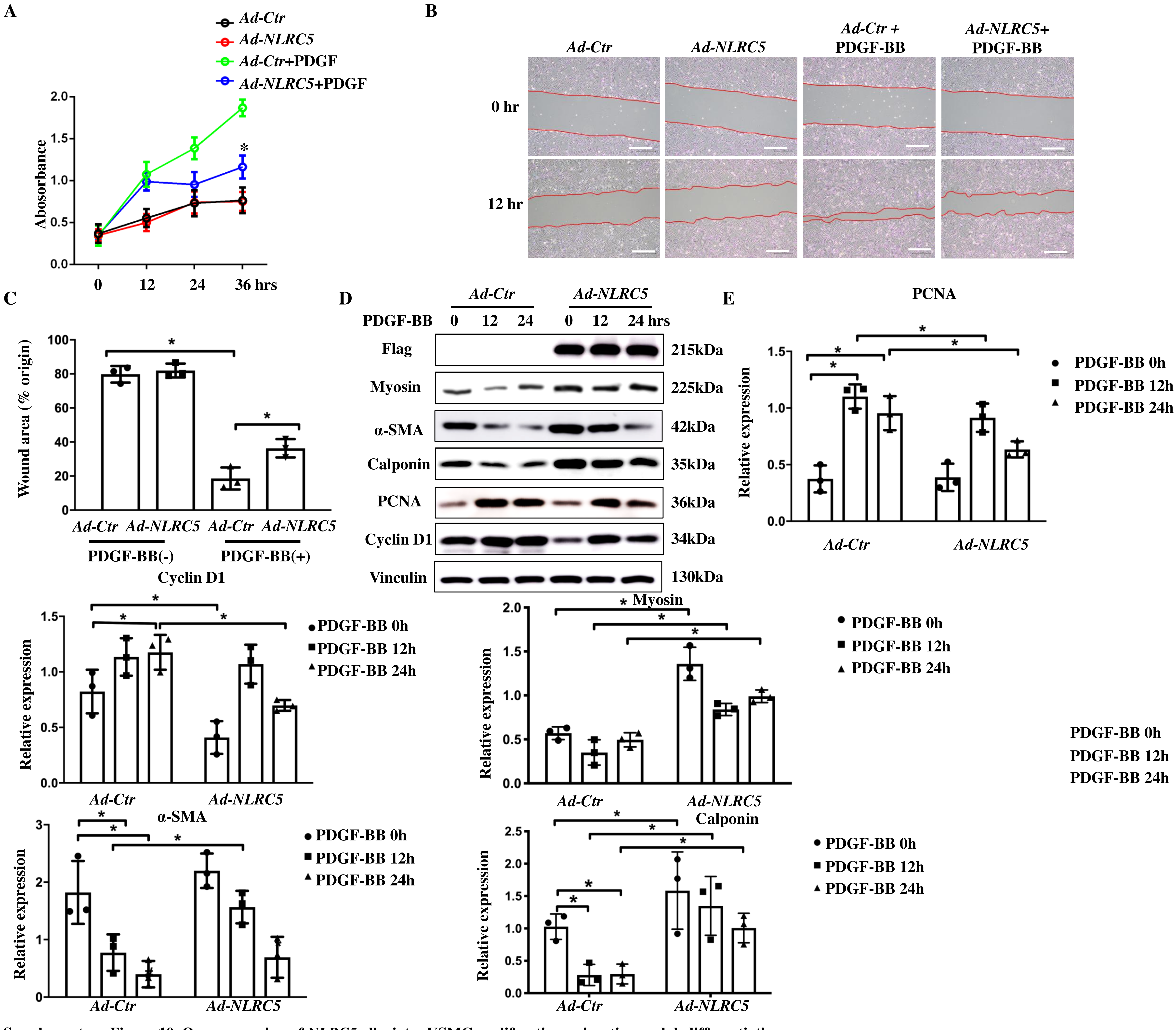

Supplementary Figure 10. Overexpression of *NLRC5* alleviates VSMC proliferation, migration and dedifferentiation.

A. The proliferation of human smooth muscle cells (HASMCs) transducted with Ad-Control or Ad-*NLRC5* is measured by MTS assay in the presence or absence of PDGF-BB (10 ng/ml) at the indicated time points. * P<0.05 vs Ad-Ctr+PDGF-BB group. **B and C.** The migration is assessed by scratch wound assay in HASMCs with or without PDGF-BB (10 ng/ml) stimulation for 12 hours. Scale bar: 100 μm. Wound area is analyzed by ImagePro Plus software. Data are presented as mean ± SD from three independent experiments. * P<0.05. **D and E.** Representative western blotting of Flag-tag, PCNA, Cyclin D1, α-SMA, Myosin, Calponin and Vinculin in HASMCs transducted with Ad-Ctr or Ad-*NLRC5* in the presence of PDGF-BB (10 ng/ml) for 0, 12 and 24 hours. Difference across three or more groups are tested with one-way ANOVA followed by a post hoc analysis with Bonferroni test. Data are presented as mean ± SD from three independent experiments. * P<0.05. Source data are provided as a Source Data file.

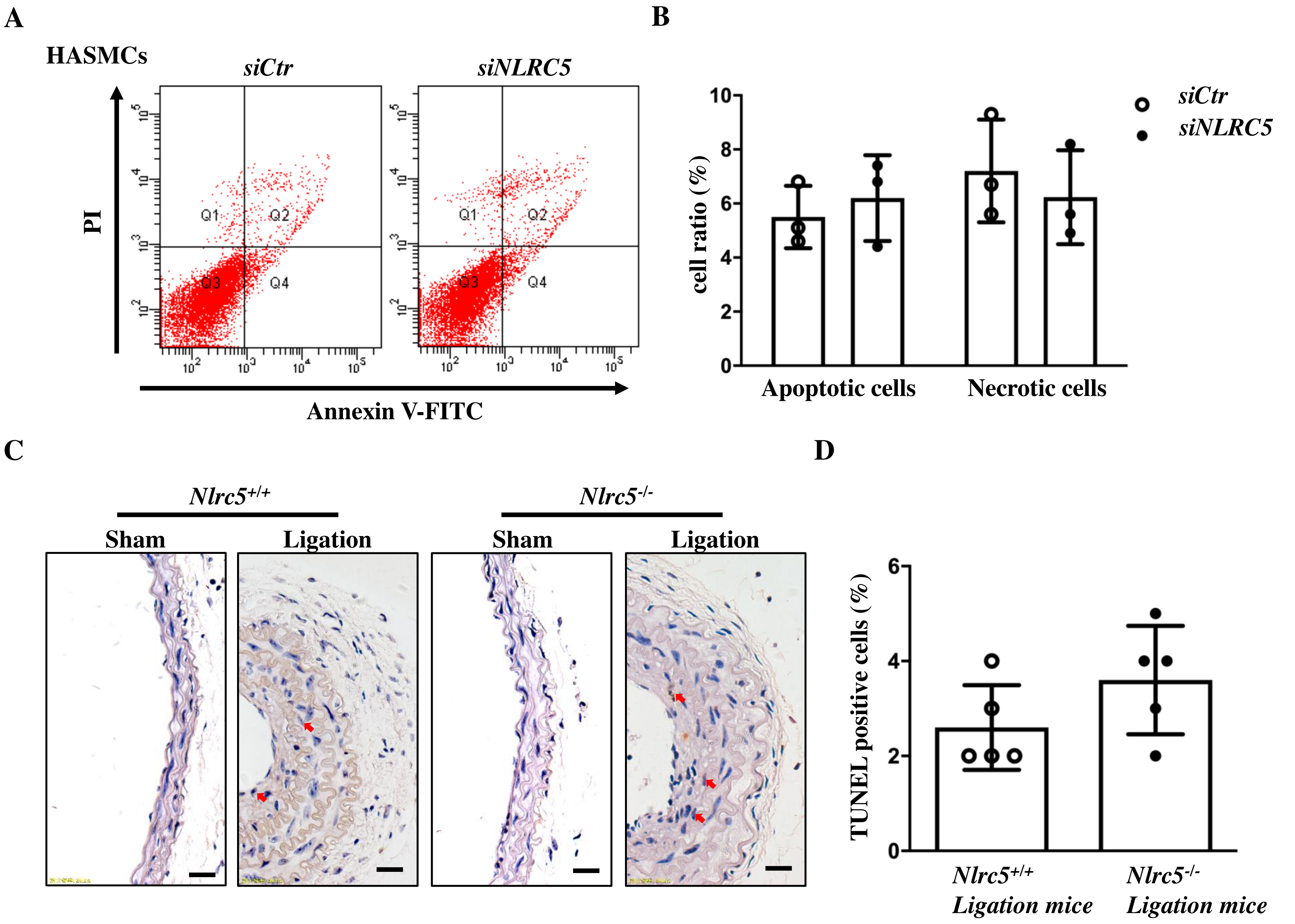

Supplementary Figure 11. Deficiency of *NLRC5* does not affect the apoptosis of smooth muscle cells after carotid ligation or starvation.

A and B. Representative flow cytometry images and quantitative results show apoptotic cell and necrotic cell ratio of serum-starved human smooth muscle cells (HASMCs) pretreated with siCtr or si*NLRC5* for 24 hours. The experiments are repeated for 3 times. **C.** Representative images of TUNEL staining of the sham and ligated carotid arteries of *Nlrc5*$^{-/-}$ and *Nlrc5*$^{+/+}$ mice. Red arrows indicate TUNEL positive cells. Scale bar: 50 μm. **D.** Quantitative results of TUNEL staining of the ligated carotid arteries of *Nlrc5*$^{-/-}$ and *Nlrc5*$^{+/+}$ mice. (n=5 per group at 3 weeks following carotid ligation). Student's t-test was used to compare two groups. Data are presented as mean ± SD. Source data are provided as a Source Data file.

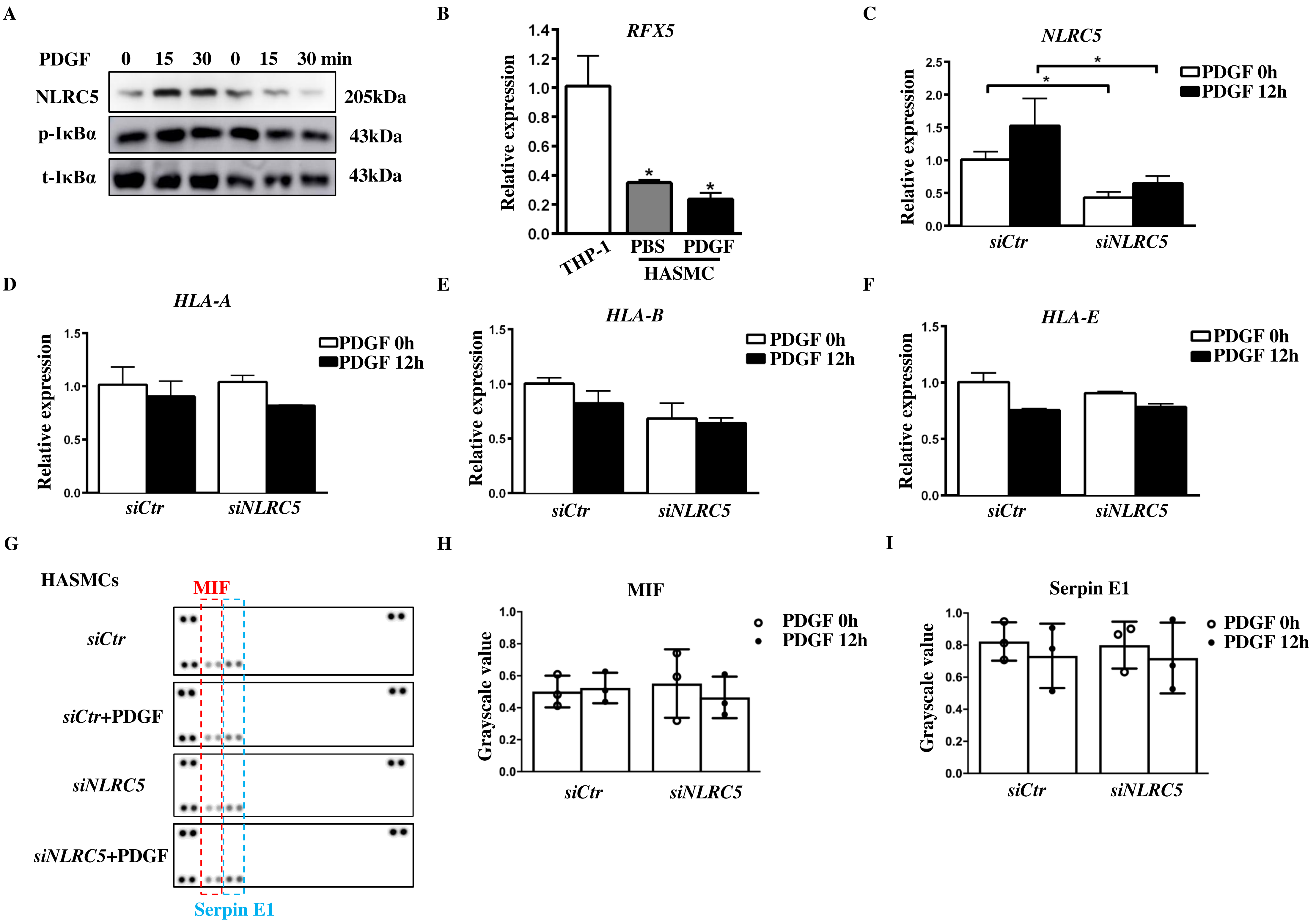

Supplementary Figure 12. Decreased *NLRC5* does not affect inflammation and HLA expression in VSMCs.
A. Western blotting of NLRC5, p-IκBα and t-IκBα in human aortic smooth muscle cells (HASMCs) transfected with siCtr or si*NLRC5* in the presence of PDGF-BB (10 ng/ml) for 0, 15 and 30 minutes. The western blots are repeated for 3 times. **B.** Expression of *Rfx5* in the monocytic cell line (THP1) and human aortic smooth muscle cells (HASMCs) with and without PDGF-BB treatment (10 ng/ml) is determined by quantitative RT-PCR. Data are presented as mean ± SD from three independent experiments. * $P<0.05$ vs THP1 group. **C-F.** *NLRC5*, *HLA-A*, *HLA-B* and *HLA-E* mRNA expression levels in HASMCs transfected with siCtr or si*NLRC5* in the presence of PDGF-BB (10 ng/ml) for 0 and 12 hours are determined by quantitative RT-PCR. Data are presented as mean ± SD from three independent experiments. * $P<0.05$. **G-I.** Human cytokine panel is applied to examine the differential expression in HASMCs transfected with siCtr or si*NLRC5* in the presence of PDGF-BB for 0 and 12 hours (10 ng/ml). Quantitative analysis is performed to evaluate the gray values of the differential expression gene MIF and Serpin E1 among groups. Data are presented as mean ± SD from three independent panels. * $P<0.05$. Source data are provided as a Source Data file.

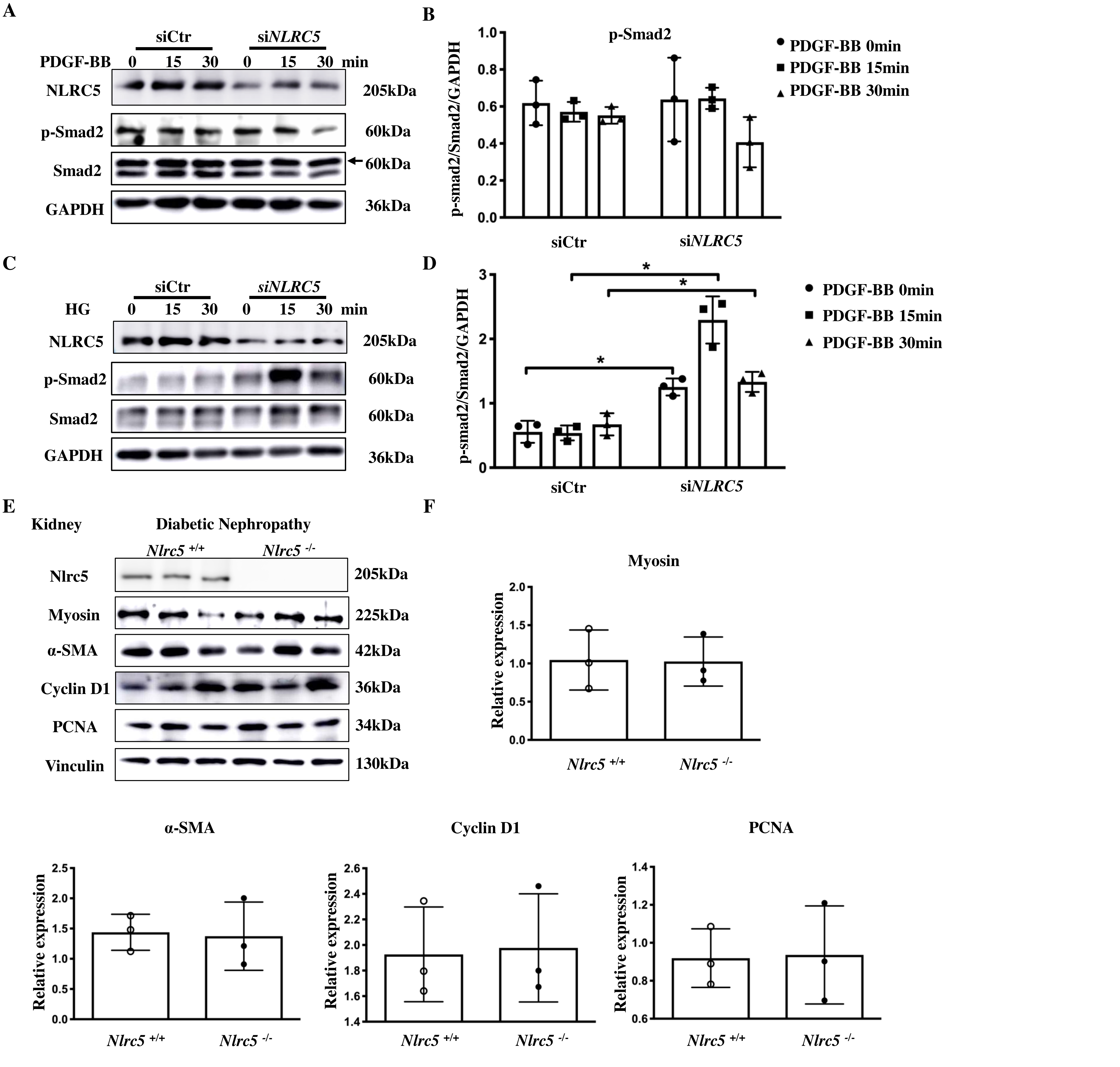

Supplementary Figure 13. Smad2 phosphorylation is stimulated by high glucose but not by PDGF-BB in HASMCs.

A and B. Western blotting of NLRC5, p-Smad2 and Smad2 in human aortic smooth muscle cells (HASMCs) transfected with siCtr or si*NLRC5* in the presence of PDGF-BB (10 ng/ml) for 0, 15 and 30 minutes. The western blots are repeated for 3 times. **C and D.** Western blotting of NLRC5, p-Smad2 and Smad2 in human aortic smooth muscle cells (HASMCs) transfected with siCtr or si*NLRC5* in the presence of high glucose (30 mM) for 0, 15 and 30 minutes. The western blots are repeated for 3 times. Difference across three or more groups are tested with one-way ANOVA followed by a post hoc analysis with Bonferroni test. **E and F.** Western blotting of Nlrc5, Myosin, α-SMA, PCNA and Cyclin D1 in diabetic kidneys of *Nlrc5*$^{+/+}$ and *Nlrc5*$^{-/-}$ mice. Data are presented as mean ± SD from three independent panels. * P<0.05. Source data are provided as a Source Data file.

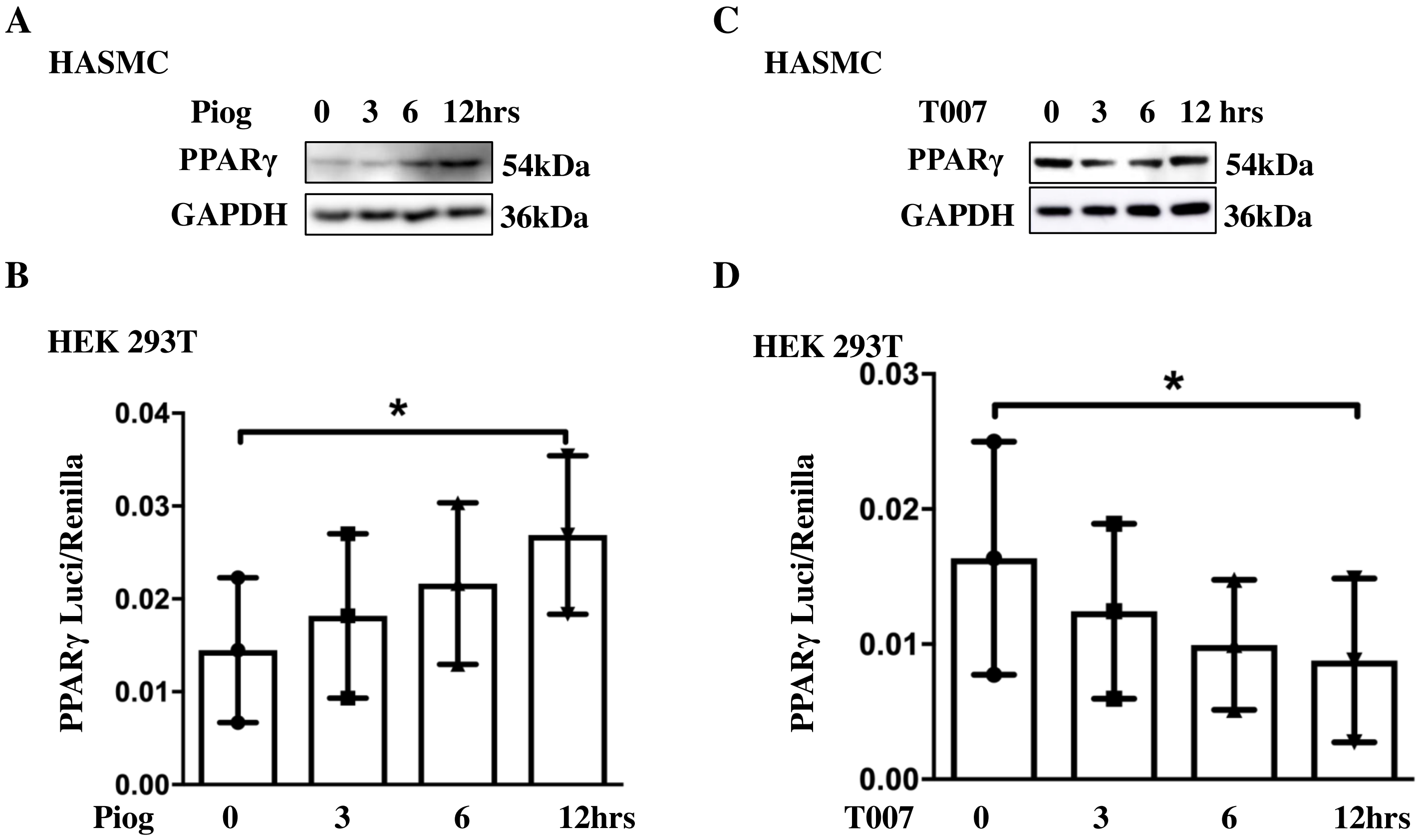

Supplementary Figure 14. Effects of pioglitazone and T0070907 on PPARγ expression and activity.
A. Western blotting of PPARγ in human aortic smooth muscle cells (HASMCs) incubated with PPARγ agonist pioglitazone (10 nM) for 0, 3, 6 and 12 hours. The western blots are repeated for 3 times. **B.** HEK293T cells transfected with PPARγ Cignal Reporter are incubated with pioglitazone (10 nM) for 0, 3, 6 and 12 hours, and analyzed for luciferase activity (fold changes relative to Renilla activity). Data are presented as mean ± SD from three independent experiments. * *P*<0.05. **C.** Western blotting of PPARγ in HASMCs incubated with PPARγ antagonist T0070907 (100 nM) for 0, 3, 6 and 12 hours. The western blots are repeated for 3 times. **B.** HEK293T cells transfected with PPARγ Cignal Reporter are incubated with T0070907 (100 nM) for 0, 3, 6 and 12 hours, and analyzed for luciferase activity (fold changes relative to Renilla activity). Data are presented as mean ± SD from three independent experiments. * *P*<0.05. Source data are provided as a Source Data file.

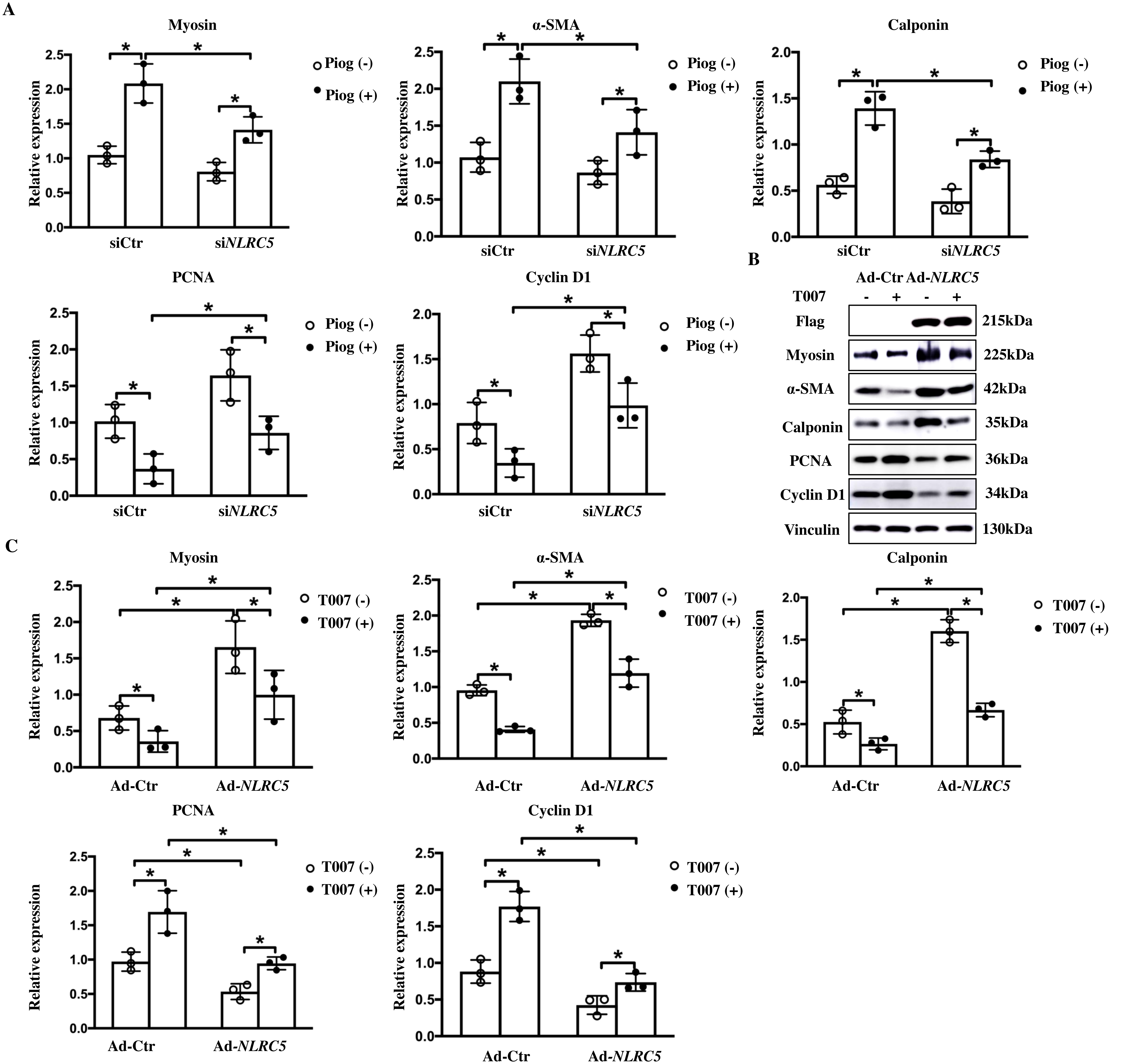

Supplementary Figure 15. Effects of pioglitazone and T0070907 on HASMC phenotype.
A. Quantification of Myosin, α-SMA, Calponin, PCNA and Cyclin D1 levels in human aortic smooth muscle cells (HASMCs) transfected with siCtr or si*NLRC5* in the presence of pioglitazone (10 nM) for 0 and 12 hours. Data are presented as mean ± SD from three independent experiments. * $P<0.05$. **B and C.** Western blotting of Flag-tag, PCNA, Cyclin D1, α-SMA, Myosin, Calponin and Vinculin in HASMCs transducted with Ad-Ctr or Ad-*NLRC5* in the presence of T0070907 (100 nM) for 0 and 12 hours. Student's t-test was used to compare two groups. Data are presented as mean ± SD from three independent experiments. * $P<0.05$. Source data are provided as a Source Data file.

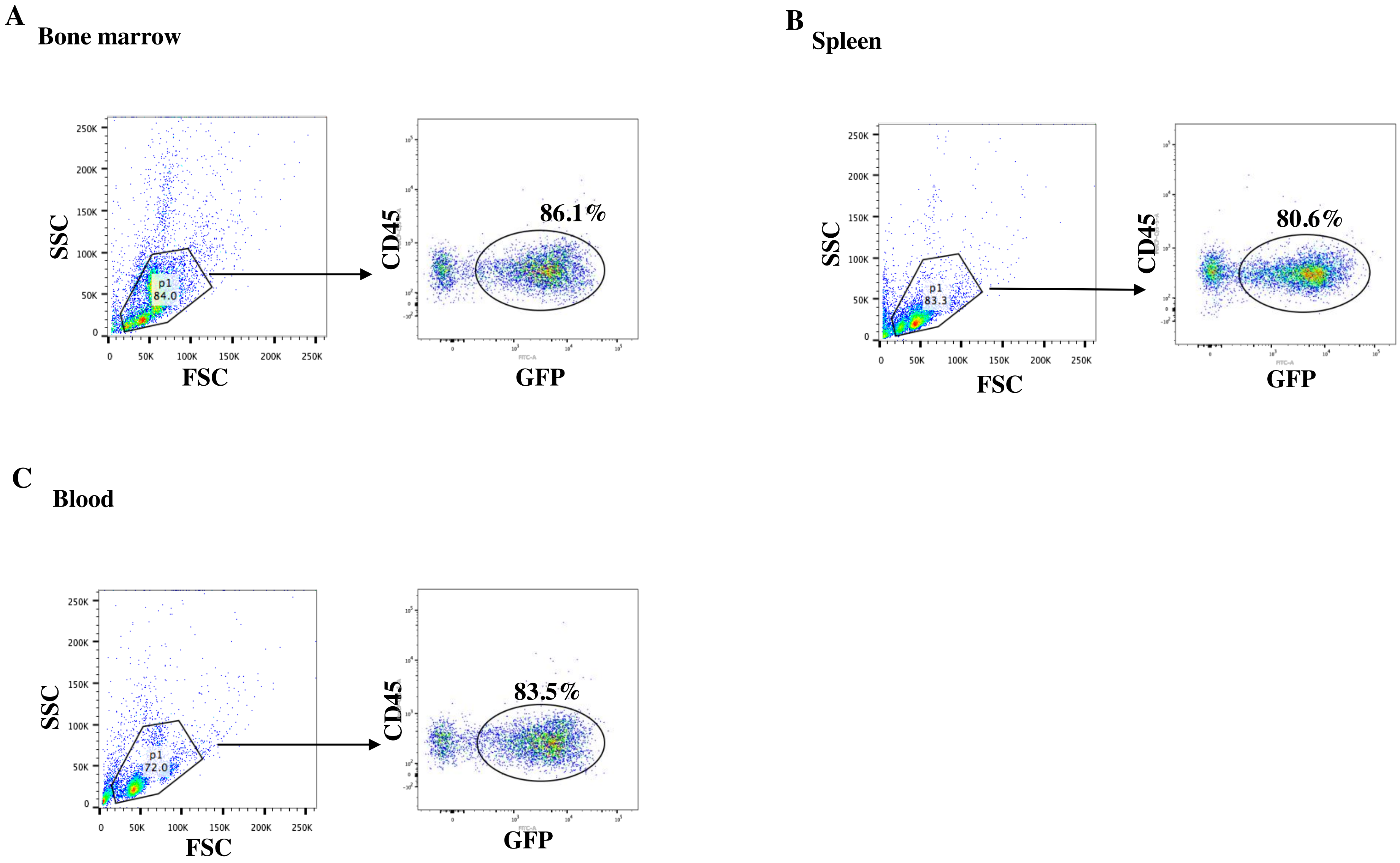

Supplementary Figure 16. Identification of C57BL/6 recipient harboring GFP transgenic bone marrow cells by flow cytometry. Gating strategy to determine the percentage of $CD45^{+}$ and GFP^{+} cells in bone marrow (**A**) spleen (**B**) and peripheral blood (**C**) of C57BL/6 recipients (n=3) harboring GFP transgenic bone marrow cells.

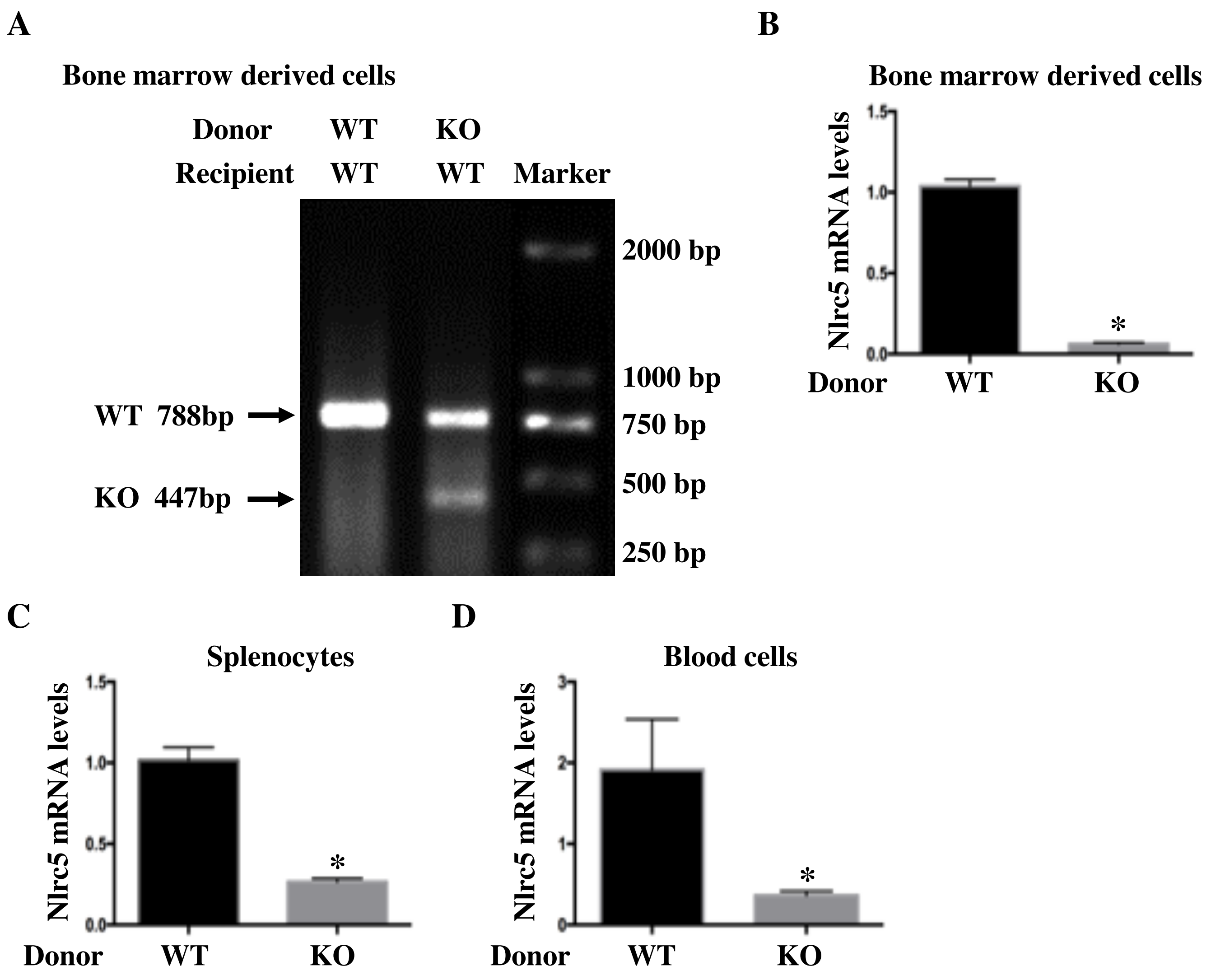

Supplementary Figure 17. Lethally-irradiated wild-type (WT) mice received bone marrow transplantation (BMT) from WT or *Nlrc5*$^{-/-}$ (KO) donors followed by 3 weeks recovery.

A. Genotyping of BM derived cells from WT recipients is performed to verify the efficiency of BMT. Lane 1 shows genotyping in WT mice received BMT from WT donor. Lane 2 shows genotyping in WT mice received BMT from KO donor. Lane 3 shows the electrophoresis marker. **B-D.** Quantitative PCR is performed to verify the efficiency of BMT in bone marrow derived cells **(B)**, splenocytes **(C)** and peripheral blood cells **(D)** from WT recipients. (n=5 per group at 3 weeks following BMT). Student's t-test was used to compare two groups. Data are presented as mean ± SD from three independent experiments. * $P<0.05$. Source data are provided as a Source Data file.

Supplementary Table 1. The baseline characteristics of the patients.

	Healthy control (n=3)	Coronary artery disease (n=3)
Age	64.3 ± 9.3	65.5 ± 9.5
Male	2 (66.7%)	2 (66.7%)
Hypertension	2 (66.7%)	2 (66.7%)
Diabetes	0 The baseline characteristics of the patients.	1 (33.3%)
Hyperlipidemia	1 (33.3%)	1 (33.3%)

Supplementary Table 2. Summary of fluorescently labelled antibodies.

Antibody	Catalog No.	Dilution ratio
**FITC rat anti-mouse CD4**	553046, BD Biosciences	1:100
**PerCP-Cy™5.5 rat anti-mouse CD8a**	551162, BD Biosciences	1:100
**PerCP rat anti-mouse CD45**	557235, BD Biosciences	1:100
**PE rat anti-mouse CD11b**	557397, BD Biosciences	1:100
**APC rat anti-mouse Ly6G and Ly-6C**	553129, BD Biosciences	1:100

Supplementary Table 3. Summary of primer sequences used for RT-PCR and ChIP.

Gene (Human)	Sequence	
NLRC5	Forward:	5’ GCTCGGCAACAAGAACCTGT 3’
	Reverse:	5’ GGTCCAAGGTCTCGTTCCT 3’
HLA-A	Forward:	5’ AAAAGGAGGGAGTTACACTCAGG 3’
	Reverse:	5’ GCTGTGAGGGACACATCAGAG 3’
HLA-B	Forward:	5’ CAGTTCGTGAGGTTCGACAG 3’
	Reverse:	5’ CAGCCGTACATGCTCTGGA 3’
HLA-E	Forward:	5’ TTCCGAGTGAATCTGCGGAC 3’
	Reverse:	5’ GTCGTAGGCGAACTGTTCATAC 3’
CD36	Forward:	5' AGA TGC AGC CTC ATT TCC AC 3'
	Reverse:	5' GCC TTG GAT GGA AGA ACA AA 3'
AP2	Forward:	5' AAC CTT AGA TGG GGG TGT CC 3'
	Reverse:	5' GTG GAA GTG ACG CCT TTC AT 3'
CITED2	Forward:	5' TTT CAA CCA TCA CCC CTA CC 3'
	Reverse:	5' CTG GTT TGT CCC GTT CAT CT 3'
GAPDH	Forward:	5’ GGA GCG AGA TCC CTC CAA AAT 3’
	Reverse:	5’ GGC TGT TGT CAT ACT TCT CAT GG 3’
NLRC5-ChIP	Forward:	5’ CTC CAC GAG TGC CAG AC 3’
	Reverse:	5’ CAG GGA AGC AGC TCA TGT TC 3’
