# Supplementary material for: NLRC5 inhibits neointima formation following vascular injury and directly interacts with PPARγ
Source: Nat Commun. 2019 Jun 28;10:2882. doi: 10.1038/s41467-019-10784-y (PMC6599027; doi:10.1038/s41467-019-10784-y)
Supplement: Supplementary file 3 — Reporting Summary [file 41467_2019_10784_MOESM3_ESM.pdf]

nature research

Corresponding author(s): Jianhui Zhuang, Yawei Xu and Wenhui Peng

Last updated by author(s): Feb 2, 2019

Reporting Summary

Nature Research wishes to improve the reproducibility of the work that we publish. This form provides structure for consistency and transparency in reporting. For further information on Nature Research policies, see Authors & Referees and the Editorial Policy Checklist.

Statistics

For all statistical analyses, confirm that the following items are present in the figure legend, table legend, main text, or Methods section.

n/a	Confirmed	
☐	☒	The exact sample size (n) for each experimental group/condition, given as a discrete number and unit of measurement
☐	☒	A statement on whether measurements were taken from distinct samples or whether the same sample was measured repeatedly
☐	☒	The statistical test(s) used AND whether they are one- or two-sided<br>*Only common tests should be described solely by name; describe more complex techniques in the Methods section.*
☐	☒	A description of all covariates tested
☐	☒	A description of any assumptions or corrections, such as tests of normality and adjustment for multiple comparisons
☐	☒	A full description of the statistical parameters including central tendency (e.g. means) or other basic estimates (e.g. regression coefficient) AND variation (e.g. standard deviation) or associated estimates of uncertainty (e.g. confidence intervals)
☐	☒	For null hypothesis testing, the test statistic (e.g. F, t, r) with confidence intervals, effect sizes, degrees of freedom and P value noted<br>*Give P values as exact values whenever suitable.*
☒	☐	For Bayesian analysis, information on the choice of priors and Markov chain Monte Carlo settings
☒	☐	For hierarchical and complex designs, identification of the appropriate level for tests and full reporting of outcomes
☒	☐	Estimates of effect sizes (e.g. Cohen's d, Pearson's r), indicating how they were calculated

Our web collection on statistics for biologists contains articles on many of the points above.

Software and code

Policy information about availability of computer code

Data collection	ZEN 2009 light edition (Carl Zeiss), BD FACSDiva, Light Cycler 96 version 1.1
Data analysis	GraphPad Prism version 6.0, FlowJo version 10, Microsoft Excel version 16.14, Image-Pro Plus 6.0

For manuscripts utilizing custom algorithms or software that are central to the research but not yet described in published literature, software must be made available to editors/reviewers. We strongly encourage code deposition in a community repository (e.g. GitHub). See the Nature Research guidelines for submitting code & software for further information.

Data

Policy information about availability of data

All manuscripts must include a data availability statement. This statement should provide the following information, where applicable:

- Accession codes, unique identifiers, or web links for publicly available datasets
- A list of figures that have associated raw data
- A description of any restrictions on data availability

The structural domains of NLRC5 are defined according to the structural database information (UniProtKB-Q86WI3, NLRC5_HUMAN, https://www.uniprot.org/uniprot/Q86WI3#structure). The base sequence representing the consensus PPARγ binding motif is acquired from JASPAR (jaspar.genereg.net). All the data supporting the findings of this study are available within the article and its Supplementary Information files and from the corresponding author upon reasonable request. The source data underlying all Figures and Supplementary Figures are provided as a Source Data file. A reporting summary for this article is available as a Supplementary Information file.

Field-specific reporting

Please select the one below that is the best fit for your research. If you are not sure, read the appropriate sections before making your selection.

☒ Life sciences ☐ Behavioural & social sciences ☐ Ecological, evolutionary & environmental sciences

For a reference copy of the document with all sections, see nature.com/documents/nr-reporting-summary-flat.pdf

Life sciences study design

All studies must disclose on these points even when the disclosure is negative.

Sample size	Sample size was selected based on the number of patients for whom we had gathered complete data sets.
Data exclusions	Not applicable
Replication	All attempts for replication of the results, where applicable, were successful.
Randomization	not applicable
Blinding	not applicable

Reporting for specific materials, systems and methods

We require information from authors about some types of materials, experimental systems and methods used in many studies. Here, indicate whether each material, system or method listed is relevant to your study. If you are not sure if a list item applies to your research, read the appropriate section before selecting a response.

Materials & experimental systems

n/a	Involved in the study	
☐	☒	Antibodies
☐	☒	Eukaryotic cell lines
☒	☐	Palaeontology
☐	☒	Animals and other organisms
☐	☒	Human research participants
☒	☐	Clinical data

Methods

n/a	Involved in the study	
☒	☐	ChIP-seq
☐	☒	Flow cytometry
☒	☐	MRI-based neuroimaging

Antibodies

Antibodies used	The following antibodies were used for immunofluorescence and immunohistochemistry staining: NLRC5 (ab105411, Abcam, 1:100 dilution), α-smooth muscle actin (ab7817, Abcam, 1:100 dilution), PPARγ (sc-7196, Santa Cruz, 1:50 dilution), CD31 (557355, BD Bioscience, 1:100 dilution), FLAG (ab49763, Abcam, 1:100 dilution), myc (2276, Cell Signaling Technology, 1:100 dilution) and PCNA (sc-25280, Santa Cruz, 1:50 dilution). The following anti-mouse antibodies were from BD Biosciences and used for flow cytometry: CD4-FITC (553046, 1:100 dilution), CD8a-PerCP-Cy™5.5 (551162, 1:100 dilution), CD45-PerCP (557235, 1:100 dilution), CD11b-PE (557397, 1:100 dilution), Ly6G and Ly6C-APC(553129, 1:100 dilution). The following antibodies were used for western blot: PPARγ (sc-7196, Santa Cruz, 1:400 dilution), RXRα (3085, Cell Signaling Technology, 1:1000 dilution), PCNA (ab29, Abcam, 1:1000 dilution), Cyclin D1 (2978, Cell Signaling Technology, 1:1000 dilution), α-SMA (ab5694, Abcam, 1:1000 dilution), Calponin (ab46794, Abcam, 1:1000 dilution), Myosin (ab53219, Abcam, 1:2000 dilution), NLRC5 (ab117624, Abcam, 1:500 dilution), GAPDH (60004-1-Ig, Proteintech, 1:10000 dilution), Lamin B1 (66095-1-Ig, Proteintech, 1:3000 dilution), α-tubulin (ab52866, Abcam, 1:5000 dilution), β-actin (60008-1-Ig, Proteintech, 1:3000 dilution), vinculin (sc73614, Santa Cruz, 1:5000 dilution), FLAG (ab1162, Abcam, 1:1000 dilution) and myc (2276, Cell Signaling Technology, 1:1000 dilution).
Validation	Validation was done by the vendor

Eukaryotic cell lines

Policy information about cell lines

Cell line source(s)	Human aortic smooth muscle cells (HASMCs) were from ScienCell Research Laboratories. HEK293T cells were from ATCC.
Authentication	None of the cell lines used were authenticated.

Mycoplasma contamination	All tested negative for mycoplasma contamination.
Commonly misidentified lines (See ICLAC register)	N/A

Animals and other organisms

Policy information about studies involving animals; ARRIVE guidelines recommended for reporting animal research

Laboratory animals	For laboratory animals, we used male Nlrc5 knockout (on C57BL/6 Background) and littermate wild type mice.
Wild animals	The study did not involve wild animals.
Field-collected samples	The study did not involve samples collected from the field.
Ethics oversight	Animal procedures were approved by the Animal Care and Use Committees of Shanghai Tenth People's Hospital.

Note that full information on the approval of the study protocol must also be provided in the manuscript.

Human research participants

Policy information about studies involving human research participants

Population characteristics	There were three healthy control and three patients with coronary artery disease respectively. Two of the healthy control patients were male and the average age was 64.3± 9.3. Among them, two were previously diagnosed as hypertension and one was diagnosed as hyperlipidemia. Two of the patients with coronary artery disease were male and the average age was 65.5 ± 9.5. Among them, two were previously diagnosed as hypertension and one was diagnosed as hyperlipidemia and diabetes.
Recruitment	Healthy coronary arteries were obtained from three patients undergoing trauma surgery without coronary plaques. Human coronary plaques were obtained from three patients undergoing coronary artery bypass grafting. The written informed consent was collected from each patient and/or their relatives.
Ethics oversight	The study was approved by the Ethical Committee of Shanghai Tenth People's Hospital.

Note that full information on the approval of the study protocol must also be provided in the manuscript.

Flow Cytometry

Plots

Confirm that:

☒ The axis labels state the marker and fluorochrome used (e.g. CD4-FITC).

☒ The axis scales are clearly visible. Include numbers along axes only for bottom left plot of group (a 'group' is an analysis of identical markers).

☒ All plots are contour plots with outliers or pseudocolor plots.

☒ A numerical value for number of cells or percentage (with statistics) is provided.

Methodology

Sample preparation	Peripheral blood cells from each mouse were obtained using heparin anticoagulant tubes before harvesting other tissues. Mice were then perfused with ice-cold PBS thoroughly before spleens, and tibia, and femur bones were harvested. Bone marrow cells from tibia and femur bones were flushed out using cold Roswell Park Memorial Institute (RPMI)-filled syringe, whereas cells were isolated from spleens using mesh. The suspensions of bone marrow cells or splenocytes were obtained after going through a 0.45 μm strainer, and red blood cells were lysed in the dark (10×FACS Lysing Solution, BD Pharmingen, USA). Cell suspensions were blocked with 1% BSA solution for 15 minutes at 4℃ and then stained with corresponding fluorescently labelled antibodies, diluted in 0.1% BSA solution at the indicated concentration. The following antibodies were used for flow cytometry: FITC rat anti-mouse CD4 (553046, BD Biosciences), PerCP-Cy™5.5 rat anti-mouse CD8a (551162, BD Biosciences), PerCP rat anti-mouse CD45 (557235, BD Biosciences), PE rat anti-mouse CD11b (557397, BD Biosciences), APC rat anti-mouse Ly6G and Ly-6C (553129, BD Biosciences). The antibodies were summarized in Supplementary Table II.
Instrument	BD LSR-II
Software	FlowJo (V10.0.7, USA)
Cell population abundance	Viable cells in all analyses were over 85% based on fsc/ssc. GFP+ cells were over 80% purity in bone marrow, spleen and blood as detected by FACS. CD45+ cells were over 85% purity in spleen and blood and 75% in bone marrow.
Gating strategy	The Fluorescence Minus One Control were used to define the boundaries between positive and negative cell populations in mutiple fluorochromes panels. Viable cells were selected based on fsc/ssc. The following gating strategies were used: CD4 T cells were gated as CD4+. CD8 T cells were gated as CD8a+. Myeloid cells were gated as CD45+CD11b+Ly6G and Ly6C+. To identify

GFP positive cells, cells were labelled with PerCP rat anti-mouse CD45 antibody and the positive cells were gated as PerCP high and FITC high.

☒ Tick this box to confirm that a figure exemplifying the gating strategy is provided in the Supplementary Information.
